# Assessment of Vulnerability to Extreme Flash Floods in Design Storms

**DOI:** 10.3390/ijerph8072907

**Published:** 2011-07-14

**Authors:** Eung Seok Kim, Hyun Il Choi

**Affiliations:** 1 Department of Civil Engineering, Sunmoon University, 100, Kalsan-ri, Tangjeong-myeon, Asan-si, Chungnam-do, 336-708, Korea; E-Mail: hydrokes@sunmoon.ac.kr; 2 Department of Civil Engineering, Yeungnam University, 214-1, Dae-dong, Gyeongsan-si Gyeongbuk-do, 712-749, Korea

**Keywords:** flash flood index, extreme flood, design storm, runoff hydrograph

## Abstract

There has been an increase in the occurrence of sudden local flooding of great volume and short duration caused by heavy or excessive rainfall intensity over a small area, which presents the greatest potential danger threat to the natural environment, human life, public health and property, *etc*. Such flash floods have rapid runoff and debris flow that rises quickly with little or no advance warning to prevent flood damage. This study develops a flash flood index through the average of the same scale relative severity factors quantifying characteristics of hydrographs generated from a rainfall-runoff model for the long-term observed rainfall data in a small ungauged study basin, and presents regression equations between rainfall characteristics and the flash flood index. The aim of this study is to develop flash flood index-duration-frequency relation curves by combining the rainfall intensity-duration-frequency relation and the flash flood index from probability rainfall data in order to evaluate vulnerability to extreme flash floods in design storms. This study is an initial effort to quantify the flash flood severity of design storms for both existing and planned flood control facilities to cope with residual flood risks due to extreme flash floods that have ocurred frequently in recent years.

## 1. Introduction

A flash flood is local flooding of great volume and short duration. Such sudden local floods have occured quite frequently in recent years due to heavy or excessive rainfall in a short period of time over a small area. The Korea Meteorological Administration (KMA) reported that as there has been an increase in summer precipitation and localized torrential downpours, the yearly precipitation has also increased approximately 19% in the 1912–2008 period. Since the temporal and spatial fluctuations of the precipitation are expected to worsen, the climatic and geomorphic vulnerability of watersheds in the Korean Peninsula is exposed to flash flood hazards caused by localized convective storms of short duration over small steep slope regions. The rapid runoff associated with debris flow has inundated some watershed areas and the river flow altered by debris flow has resulted in some flood damage such as bank erosion and bridge collapses, as reported in the annual natural disaster bulletin [1]. A rapid local flood poses the greatest potential danger threat to human life, public health and property, the natural environment and ecosystems, water and other natural resources, etc. Although such types of dangerous flood damage now constitute national-wide natural disasters, the structural and non-structural alternative plans for flood mitigation have been mainly carried out for large basins in Korea. The Fourth Assessment Report (AR4) of the United Nations Intergovernmental Panel on Climate Change (IPCC) [2] identified the following key priorities which must be addressed to narrow gaps between current knowledge, and policymaking needs; quantitative assessment of the sensitivity, adaptive capacity, and vulnerability of natural and human systems to climate change, particularly changes in the range of climatic variation and the frequency and severity of extreme climate events. Therefore, it is necessary to assess flood mitigation ability or vulnerability of flood control facilities and to devise both structural and non-structural flood mitigation measures against the residual risk of the extreme floods in ungauged small watersheds.

Flash floods have been studied mostly as climatological phenomena, especially focused on the temporal and spatial characteristics of rainfall [3–5]. From the hydrological perspective, there have been several studies on the characterization of flash floods by analysis of the characteristics of runoff hydrographs. Kyiamah [6] was the first to characterize flash floods from a runoff perspective using runoff hydrographs. Bhaskar *et al*. [7] presented a flash flood index using runoff hydrograph characteristics such as the rising curve gradient, flood magnitude ratio, and flood response time evaluated directly from observed runoff hydrographs of 30 flood events from four watersheds in eastern Kentucky. Jung [8] estimated the flash flood index for several flood events of the Bo-chung river basin in Korea following Bhaskar *et al*. [7]. In these studies, the flash flood index was determined by the sum of the three relative severity factors, each using a different ordinal scale where class intervals are to some extent arbitrary. Kim and Kim [9] estimated the flash flood index for investigating the relative severity of flash floods in the Han River basin with 101 flood events, and quantified the flash flood severity for some flood events caused by heavy rainfall in July of 2006. However, there was no attempt to quantify the severity of floods occurred in small catchments where usually observations are not available. Therefore, this study has modified the flash flood index presented by Bhaskar *et al*. [7] and developed a new flash flood index determined by the average of relative severity factors on the same scale ratio to the recorded maximum value. IPCC AR4 [2] pointed out that we need to focus on improvement of systems and methods for long-term monitoring and understanding the consequences of climate change and other stresses on human and natural systems. The flash flood indexing methods are implemented by quantifying the characteristics of hydrographs generated from a rainfall-runoff model, HEC-HMS (Hydrologic Engineering Center-Hydrologic Modeling System), for both the annual maximum rainfall event series during 36 years and the probability rainfall data in return periods of 2, 3, 5, 10, 20, 50, 100, and 200 years and for duration times of 1, 3, 6, 12, 18, and 24 hours in a small ungauged basin, the Oui-mi River basin in Korea. This study has provided flash flood index-duration-frequency (*FI-D-F*) relation curves developed from rainfall intensity-duration-frequency (*I-D-F*) relations for the use of evaluating vulnerability to extreme flood conditions in a design storm in order to establish disaster countermeasures for residual flood risk in both existing and planned flood control facilities.

## 2. Study Catchment

**Figure 1:**
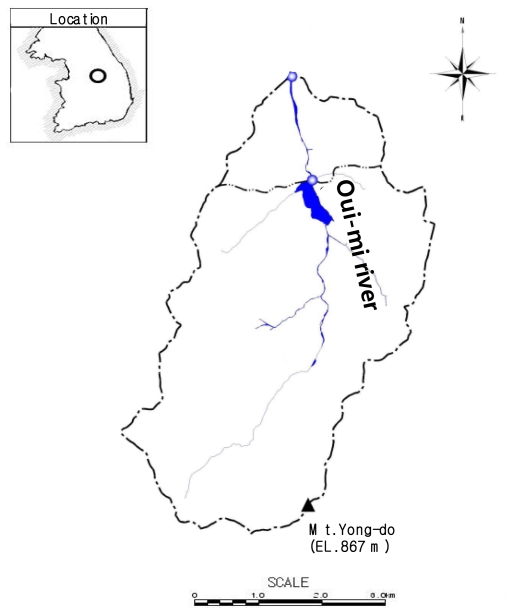
Basin map for the Oui-mi River.

The characterization of local flash flooding was applied to a small hilly drainage catchment, the Oui-mi River basin, located between 128°10′35″E~128°11′37″E, and 37°14′39″N~37°15′29″N, as shown in Figure 1. The Oui-mi River catchment is a natural basin comprised of 86.3% of woodlands, 12.2% of farmlands, and 1.5% of other types. This study basin is 7.52 m long with a size of 16.74 km^2^, and the average elevation is 544.9 m above mean sea level, with an average slope of 53.4% [10].

There is a rainfall gauge station around the study basin, the Jae-chun Gauge Station managed by the Meteorological Administration, where long-term hourly precipitation data is available. The annual maximum rainfall data series during 1973–2008 were collected from this gauge station. The annual mean rainfall volume was 1,322.5 mm over the same period, and the recorded maximum depth of a single rainfall event is 228.5 mm in Sep. 11, 1990.

## 3. Flash Flood Indexing Method

To quantify the relative severity of flash floods in small ungauged catchments, this study determines a flash flood index, *FI*, from flood runoff hydrographs simulated by a rainfall-runoff model for the annual maximum precipitation event series in a study basin. Bhaskar *et al*. [7] has characterized the flash flood severity by defining a flash flood index evaluated directly from the observed flood hydrograph characteristics such as the rising curve gradient, flood magnitude ratio, and flood response time. This flash flood index from Bhaskar *et al*. [7] is obtained from the sum of three characteristics quantified by the relative severity factors. The problematic issue is in quantifying these relative severity factors by using each different ordinal scale of assignment where the choice of class intervals is to some extent arbitrary. Hence, this study computes all relative severity factors on the same scale ratio to each recorded maximum value. The flash flood indexes suggested in this study are determined by the average of the relative severity factors. Details of the flash flood indexing procedure are presented below.

### 3.1. Flood Runoff Hydrographs

Flood runoff hydrographs are generated from a rainfall-runoff model, HEC-HMS [11], using the annual maximum precipitation event series of the Jae-chun Gauge Station in the Oui-mi River basin for past 36 years (1973–2008). The NRCS (Natural Resources Conservation Service) curve number method is used for the loss rate method and the Clark unit hydrograph is used for the transform method. The NRCS curve number is averaged for the study basin as 70.1, and the storage coefficient is estimated as 1.18 hrs. Table 1 (column 2) shows that the maximum peak flood discharge of 183.3 m^3^/s occurs in 5 August 2007 and the minimum peak flood discharge of 14.5 m^3^/s occurs in 23 August 1974 among the 36 annual maximum simulated hydrographs in the Oui-mi River basin.

### 3.2. Rising Curve Gradient (K)

Bhaskar *et al*. [7] described the rising limb of hydrographs as an exponential equation using the rising curve gradient, *k*:

(1) $$Q_{t}=Q_{0}e^{kt}$$

where *Q*_0_ is the specified initial discharge in the rising limb of hydrographs, and *Q* *_t_* is the discharge at a later time *t* close to the time to peak. This exponential function usually used for hydrograph recession curves not only has a problem to describe the rising curve gradient in cases of double-peak hydrographs, but also has a difficulty in defining the specified initial discharge, *Q*_0_, for simulated hydrographs. Hence, this study expresses the rising limb of the simulated hydrograph using a mean slope gradient approximation:

(2) $$K=\frac{\left(Q_{p}/A\right)}{T}$$

where *K* is a mean slope gradient of the rising limb, *Q_p_* is the peak discharge, *T* is a duration time between the starting time of a flood event and the peak flow occurrence time, and *A* is the drainage area. The rising curve gradient, *K*, is computed for the specific discharge (discharge per unit area) with a unit of mm/hr^2^.

The large values of the parameter *K* can be associated with a rapid local flood of great volume because the rising curve gradient represents the steepness of the rising limb of flood hydrographs. The range of values for the rising curve gradient, *K*, is from 0.30 mm/hr^2^ to 5.63 mm/hr^2^ for the Oui-mi River basin as shown in column 4 of Table 1. The relative severity for the rising curve gradient, *K*, is quantified as a dimensionless index, *RK*, which is a ratio of each flood’s *K* *_i_* to the recorded maximum value, *K*_max_:

(3) $$RK=\frac{K_{i}}{K_{\text{max}}}$$

### 3.3. Peak Discharge Magnitude (M)

Bhaskar *et al*. [7] presented the flood magnitude ratio, *m*, which means a ratio of the peak flood discharge to the long-term average discharge:

(4) $$m=Q_{p}/Q_{a}$$

where *Q_a_* is the long-term average discharge. Because the long-term average discharge, *Q_a_*, is not only unavailable in ungauged catchments, but also canceled out later in obtaining the relative severity factor in Equation 6, the parameter *m* is replaced with the peak specific discharge magnitude, *M*, as:

(5) $$M=Q_{p}/A$$

The values of the peak discharge magnitude, *M*, varied from 3.11 mm/hr to 39.41 mm/hr for the Oui-mi river basin as shown in column 5 of Table 1. The relative severity factor, *RM*, is also computed by a ratio of each flood event’s *M_i_* to the recorded maximum value, *M*_max_ :

(6) $$RM=\frac{M_{i}}{M_{\text{max}}}$$

### 3.4. Flood Response Time (T)

The flood response time, *T,* can be measured directly from flood hydrographs. The flood response time, *T,* varied from 3 hrs to 24 hrs for the Oui-mi River basin as shown in column 6 of Table 1. Because a low value of *T* is readily associated to a high runoff velocity causing sudden local flooding, the relative severity factor, *RT*, is computed by a ratio of the inverse value of each flood event’s *T_i_* to the inverse value of the recorded minimum value, *T*_min_ :

(7) $$RT=\frac{T_{\text{min}}}{T_{i}}$$

### 3.5. Flash Flood Index

The relative severity factors need to be integrated for an overall value to evaluate flash flood severity for each flood event. Bhaskar *et al*. [7] have presented a flash flood index, *RF*, the sum of the three relative severity factors on different ordinal scale values such as *RK* =1 ~ 7, *RM* =1 ~ 16, and *RT* =1 ~ 10 where the choice of class intervals is to some extent arbitrary. Although they applied systematically each severity factor to 30 flood events from four watersheds in eastern Kentucky, the flash flood index, *RF,* determined by the sum of the three severity factors is often subjected to a certain factor with the greater scale of measurement than other factors.

This study presents a flash flood index, *RF*, by taking the average of the three relative severity factors on the same scale ratio to the recorded maximum value:

(8) $$RF=\frac{RK+RM+RT}{3}\times 100\;(\%)$$

In Equation 8, the two relative severity factors such as *RK* for the rising curve gradient and *RT* for the flood response time may represent the similar characteristics of a flood hydrograph because a low value of the flood response time can be associated with a high runoff velocity leading to the steep rising limb of flood hydrographs. This study presents another relative flood severity, *FI*, the average of the two relative severity factors, *RK* for the rising curve gradient and *RM* for the peak discharge magnitude:

(9) $$FI=\frac{RK+RM}{2}\times 100\;(\%)$$

Table 1 shows the two flash flood indexes, *RF* and *FI*, along with rainfall characteristics for the study basin.

## 4. Comparison of Flash Flood Indexes

**Figure 2:**
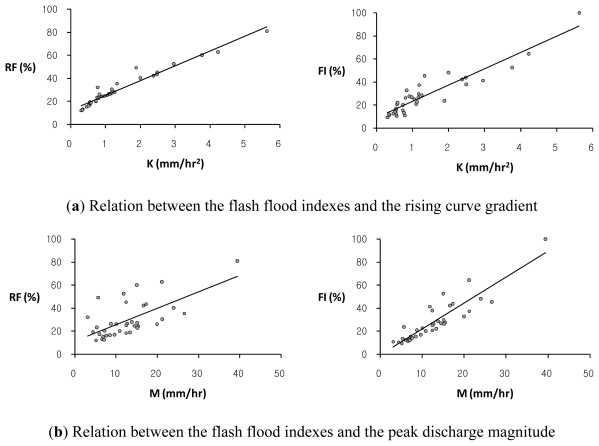
The comparison of trends between each of two flash flood indexes, *RF*, (*left*) and *FI*, (*right*) and each of two hydrograph characteristics: (**a**) the rising curve gradient, *K*; (**b**) the peak discharge magnitude, *M*, respectively, in the Oui-mi River basin.

This study examines the dependence of each of two flash flood indexes, *RF* and *FI*, on the flood hydrograph characteristics using scatter plots and regression equations as illustrated in Figure 2 and Table 2. The flash flood index, *RF*, obtained by the average of three relative severity factors such as *RK* for the rising curve gradient, *RM* for the peak discharge magnitude, and *RT* for the flood response time shows a strong dependence on the rising curve gradient, *K*, where a coefficient of determination, *R^2^*, is 0.950, while the relation between the flash flood index, *RF*, and the peak discharge magnitude, *M*, is relatively weak with *R^2^* of 0.428. The reason is because the two similar hydrograph characteristics, *K* and *T*, are in major controlling factors determining the flash flood index, *RF*, rather than the contribution of *M*. On the other hand, the flash flood index, *FI*, expressed by the average of two relative severity factors such as *RK* and *RM* is dependent at almost even level on both parameters, *K* and *M*, with *R^2^* values of 0.861 and 0.817, respectively.

**Figure 3:**
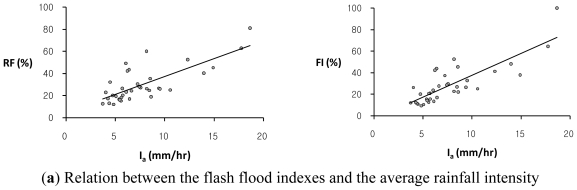

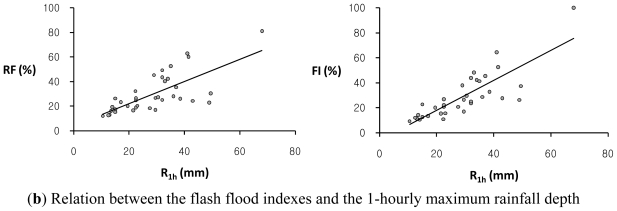
The comparison of trends between each of two flash flood indexes, *RF*, (*left*) and *FI*, (*right*) and each of two rainfall characteristics: **(a)** the average rainfall intensity, *I_a_*; **(b)** the 1-hourly maximum rainfall depth, *R_1h_*, respectively, in the Oui-mi River basin.

**Figure 5:**
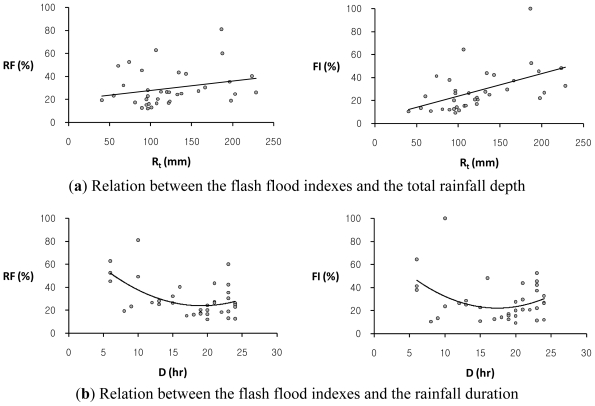
The comparison of trends between each of two flash flood indexes, *RF*, (*left*) and *FI*, (*right*) and each of two rainfall characteristics: (**a**) the total rainfall depth, *R_t_*; (**b**) the rainfall duration, *D*, respectively, in the Oui-mi River basin.

This study has also investigated the relationship between flash flood indexes, *RF* and *FI*, and several rainfall characteristics such as the average rainfall intensity, *I*_a_, the maximum rainfall depths for the 1-hour, 2-hour, and 3-hour durations, *R_1h_*, *R_2h_*, and *R_3h_*, respectively, the total rainfall depth, *R_t_*, and the rainfall duration, *D* for 36 annual maximum rainfall event series in the study basin. The average rainfall intensity refers to the total amount of rainfall for a storm event divided by the duration of the storm. The scatter plots of each of two flash flood indexes, *RF* and *FI*, versus each rainfall characteristic in the study basin are illustrated in Figure 3–Figure 5. Table 3 shows the regression equations between each rainfall characteristic and each of two flash flood indexes, *RF* and *FI*, in the study basin.

Overall, the flash flood index, *FI*, shows a much stronger relation to some rainfall data with relatively high coefficients of determination, *R^2^*, as compared with the relationship of the flash flood index, *RF*, to rainfall characteristics. The flash flood index, *FI*, which avoids double-counted relative severity factors with similar characteristics is adequate to estimate the relative flood severity in this study basin. It is observed that the Oui-mi River basin has a high linear relation between the flash flood index, *FI*, and the 3-hourly maximum rainfall depth, *R_3h_*, with the coefficient of determination, *R^2^* of 0.860, while the total rainfall amount, *R_t_*, and duration, *D*, show no evident relation to both flash flood indexes, *RF* and *FI*, as illustrated in Figure 5. This result supports the notion that local flash floods in small watersheds are mainly caused by heavy or excessive rainfall in a short period of time.

**Figure 6:**
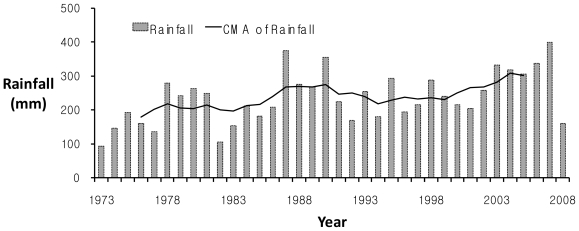
Interannual variation of the rainy season rainfall depth during 1973–2008 in the Oui-mi River basin.

**Figure 7:**
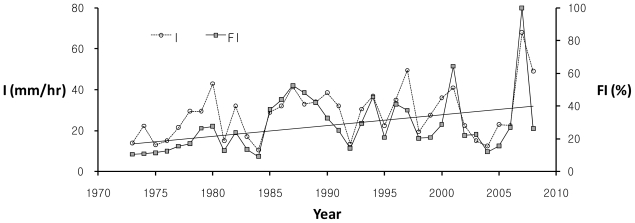
Interannual variation of the rainfall intensity, *I*, and the flash flood index, *FI*, during 1973–2008 in the Oui-mi River basin.

Figure 6 illustrates long-term trends of the averaged rainfall depth in the study watershed for the rainy season during June–September in which all annual maximum flood events for the past 36 years occur. The growing trend of the 7-year centered moving average (CMA) clearly reveals a tendency of steady increase in rainfall amounts over the years. Also, Figure 7 denotes interannual variation of the rainfall intensity, *I*, and the flash flood index, *FI*. The interannual variability of the new flash flood index, *FI*, well follows the increasing trend of the rainfall intensity for the past 36 years during 1973–2008 in this study site. Figures 6 and 7 show periodic trends that increase in annual series of both the averaged wet season rainfall amount and the maximum rainfall intensity, and Figure 7 demonstrates that the rainfall intensity can be used as a key indicator to determine and forecast the relative flash flood severity. Since it implies that flash flooding may occur more severely and frequently due to the increasing trend of the heavy rainfall intensity caused by global climate changes, the vulnerability assessment in design storms to the extreme flash flood is required to deal with current and future flooding risks.

## 5. Assessment of Vulnerability in Design Storms

Based on results showing that the rainfall intensity can capture the relative flash flood severity represented by the new flash flood index, *FI*, this study has also determined the flash flood index from probability rainfall data in the study basin to provide flash flood index-duration-frequency (*FI-D-F*) relation curves developed from rainfall intensity-duration-frequency (*I-D-F*) relation curves. *FI-D-F* curves are intended to evaluate vulnerability in a design flood to the extreme flood condition and residual flood risk of both existing and planned flood control facilities.

The Gumbel distribution is selected through estimation of parameters and goodness fit test for the several probability distributions of annual maximum rainfall data in the Oui-mi River basin. Table 4 summarizes the probability rainfall data in return periods of 2, 3, 5, 10, 20, 50, 100, and 200 years and for duration times of 1, 3, 6, 12, 18, and 24 hours, respectively. The Huff distribution [12] is used for the temporal distribution of the probability rainfall data. The Huff distribution is presented as cumulative percentages of total duration and total rainfall accumulation that consists of four quartile patterns for the bulk rainfall of the storm event as shown in Table 5. Different families of Huff distribution curves are applicable for different drainage watersheds, and the 3rd quartile is used for the design flood in the Oui-mi river basin [10].

**Figure 8:**
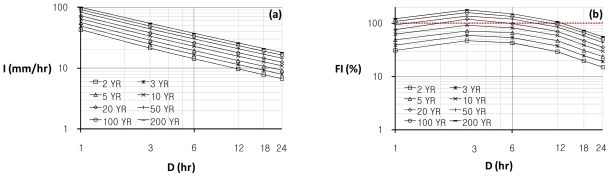
(**a**) *I-D-F* curves and (**b**) *FI-D-F* curves for the Oui-mi River basin.

The flash flood index, *FI*, is computed by hydrographs generated from a rainfall-runoff model, HEC-HMS [11], for the probability rainfall data with respect to eight return periods (2, 3, 5, 10, 20, 50, 100, and 200 yrs) and six temporal durations (1, 3, 6, 12, 18, and 24 hrs), as shown in Table 6. The flash flood index, *FI*, is determined by the average of two relative severity factors such as *RK* and *RM*, ratios of the rising curve gradient, *K*, and the peak discharge magnitude, *M*, from each design flood hydrograph to the recorded maximum values, 5.63 mm/hr^2^ of *K_max_* and 39.41mm/hr of *M_max_*, respectively. The maximum *FI* is 179.07% for a return period of 200 year and duration of three hrs, and the minimum *FI* is 14.89% for a return period of two years and duration of 24 hrs among 48 design floods (eight return periods and six temporal durations). Figure 8(a) indicates *I-D-F* curves, and (b) illustrates *FI-D-F* curves for the relation between the flash flood index, *FI*, and the rainfall duration, *D*, with respect to the eight return periods in the study basin. According to the basic plan report for the Oui-mi River maintenance works [10], some structural flood control projects are planned for the 100 year design flood in the Oui-mi River basin. In Figure 8(b), *FI* values are greater than 100% for the 100 year design storm in duration of less than 12 hrs. It means that the flood control facilities designed under these conditions, for example river levee improvement works in the Oui-mi River basin, may have countermeasure ability towards the recorded flash floods in this region. It is also implied that drainage pipe lines designed for 5 to 10 year floods may need non-structural flood mitigation plans as well as structural alternative plans in order to cope with residual flood risk to the extreme flash flood that occurs frequently in these days.

## 5. Summary and Conclusions

This study has modified the flash flood index by Bhaskar *et al*. [7], and developed a new flash flood index determined by the average of relative severity factors with the same scale ratios of each flood event’s characteristics to the recorded maximum values in order to evaluate the relative severity of floods to extreme flash floods. New relative severity factors were presented in this study to describe the characteristics of simulated runoff hydrographs for ungauged watersheds; the mean slope gradient of the rising limb and the peak specific discharge are substituted for the exponential curve gradient and the flood magnitude ratio, respectively, suggested by Bhaskar *et al*. [7]. The new flash flood indexing method was implemented by computing a dimensionless index for characteristics of hydrographs generated from a rainfall-runoff model for the long-term observed rainfall data in a small hilly ungauged catchment, the Oui-mi River basin in Korea.

The flash flood index, *FI*, the average of the two relative severity factors, *RK* and *RM*, provides a stronger relation to some rainfall characteristics as compared with the flash flood index, *RF*, including the two similar relative severity factors, *RK* and *RT*, along with *RM*. The trend between the flood flash index, *FI*, and the rainfall over a short interval, 3-hourly maximum rainfall depth, shows the best-fit line, while the flood flash index, *FI*, shows no evident relation to the total rainfall amount. It illustrates that heavy or excessive rainfall in a short period of time is a primary cause of local flash flooding in small watersheds. The best-fit regression equation between the new flash flood index, *FI,* and a certain rainfall characteristic can provide the basis database for forecasting a local flood severity directly from rainfall data in small ungauged catchments where the flood response time is quite short.

This study has also estimated the flash flood index, *FI*, from probability rainfall data with respect to eight return periods (2, 3, 5, 10, 20, 50, 100, and 200 yrs) and six temporal durations (1, 3, 6, 12, 18, and 24 hrs) in the study basin. The flash flood index, *FI*, for each design storm is determined by the two relative severity factors, *RK* and *RM* with ratios to the recorded maximum values, and then the plot of *FI-D-F* relation is developed to assess vulnerability in a design flood to extreme flash floods. *FI-D-F* curves illustrate that major flood control facilities designed for the 100 year frequency rainfall in a duration of less than 12 hrs may have flood mitigation ability towards the recorded flash floods in this study basin. However, small or midsize facilities such as drainage pipe lines designed for 5 to 10 year frequency storms may have significant vulnerability to extreme flash floods in this region. The *FI-D-F* relation curves suggested in this study is expected to be one of scientific bases for decision makers to select structural or non-structural alternative flood mitigation plans against flooding disasters. This study is an initial effort to evaluate vulnerability in design floods for both existing and planned flood control facilities in order to cope with the residual flood risk of extreme flash floods. The future study needs to incorporate various hydrometeorological perspectives, especially focused on the use of radar information, in the implementation of the new developed methodology for more precise and general flash flood predictions.

## Figures and Tables

**Figure 4:**
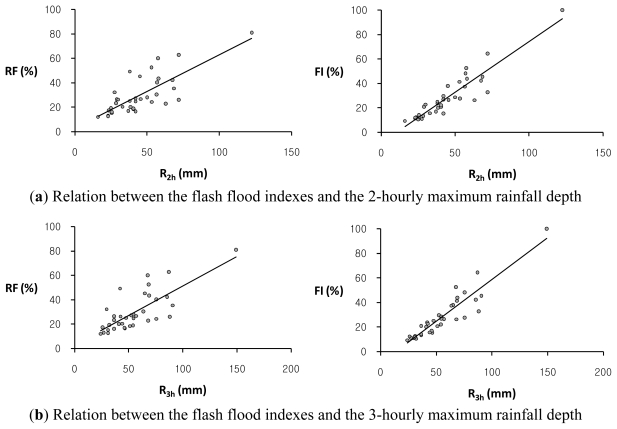
The comparison of trends between each of two flash flood indexes, *RF*, (*left*) and *FI*, (*right*) and each of two rainfall characteristics: (**a**) the 2-hourly maximum rainfall depth, *R_2h_*; (**b**) the 3-hourly maximum rainfall depth, *R_3h_*, respectively, in the Oui-mi River basin.

**Table 1:** Summary of runoff and flash flood indexing characteristics for flood events along with rainfall characteristics in the Oui-mi River basin.

No	Flood Runoff Characteristics			Flash Flood Indexing Parameters								Rainfall Characteristics					

	Flood event date	Flood peak discharge	Time to peak discharge	Rising curve gradient	Peak discharge magnitude	Flood response time	Relative Severity Factors			Flash Flood Index		Average rainfall intensity	Max. 1-hourly rainfall	Max. 2-hourly rainfall	Max. 3-hourly rainfall	Total rainfall depth	Rainfall duration time
												
	(1)	Q_p_(m^3^/s) (2)	T(hr) (3)	K(mm/hr^2^) (4)	M(mm/hr) (5)	T (hr) (6)	RK (7)	RM (8)	RT (9)	RF (%) (10)	FI (%) (11)	I_a_ (mm/hr) (12)	R_1h_ (mm) (13)	R_2h_ (mm) (14)	R_3h_ (mm) (15)	R_t_ (mm) (16)	D (hr) (17)
1	06/29/73	20.5	8.0	0.55	4.40	8.00	0.10	0.11	0.38	19.48	10.47	5.06	14.0	25.0	32.0	40.5	8
2	08/23/74	14.5	4.0	0.78	3.11	4.00	0.14	0.08	0.75	32.24	10.86	4.47	22.3	27.5	29.6	67.0	15
3	09/15/75	30.7	19.0	0.35	6.61	19.00	0.06	0.17	0.16	12.91	11.47	4.39	13.0	23.0	27.0	101.0	23
4	08/14/76	28.0	11.0	0.55	6.02	11.00	0.10	0.15	0.27	17.42	12.49	4.26	15.0	23.5	26.0	81.0	19
5	09/06/77	39.6	16.0	0.53	8.51	16.00	0.09	0.22	0.19	16.60	15.53	5.37	21.5	42.0	46.5	107.4	20
6	08/19/78	44.9	18.0	0.54	9.66	18.00	0.10	0.25	0.17	16.91	17.03	6.44	29.5	37.0	46.0	122.3	19
7	08/04/79	59.2	11.0	1.16	12.73	11.00	0.21	0.32	0.27	26.71	26.42	9.39	29.5	45.5	57.0	112.7	12
8	07/22/80	71.7	17.0	0.91	15.43	17.00	0.16	0.39	0.18	24.30	27.63	6.63	43.0	53.2	75.4	132.6	20
9	07/01/81	31.9	15.0	0.46	6.86	15.00	0.08	0.17	0.20	15.17	12.76	5.59	15.0	25.5	31.0	95.0	17
10	08/21/82	26.2	3.0	1.88	5.64	3.00	0.33	0.14	1.00	49.22	23.83	6.05	32.0	38.0	42.0	60.5	10
11	07/19/83	24.7	7.0	0.76	5.31	7.00	0.13	0.13	0.43	23.27	13.47	6.11	17.0	28.5	36.5	55.0	9
12	09/02/84	24.0	17.0	0.30	5.16	17.00	0.05	0.13	0.18	12.04	9.24	4.83	10.5	16.0	24.0	96.5	20
13	07/17/85	57.8	5.0	2.49	12.43	5.00	0.44	0.32	0.60	45.23	37.85	14.92	29.0	45.0	65.0	89.5	6
14	07/19/86	80.5	7.0	2.47	17.32	7.00	0.44	0.44	0.43	43.58	43.94	6.39	32.0	58.0	69.0	134.2	21
15	07/22/87	70.0	4.0	3.76	15.06	4.00	0.67	0.38	0.75	60.02	52.53	8.15	41.5	57.5	67.5	187.5	23
16	07/14/88	111.5	12.0	2.00	23.97	12.00	0.35	0.61	0.25	40.44	48.16	13.97	33.0	57.0	75.5	223.5	16
17	07/26/89	77.4	7.0	2.38	16.64	7.00	0.42	0.42	0.43	42.43	42.21	6.22	34.0	67.5	85.5	143.0	23
18	09/11/90	92.8	24.0	0.83	19.96	24.00	0.15	0.51	0.13	25.98	32.72	9.52	38.5	72.0	88.0	228.5	24
19	07/20/91	58.3	12.0	1.04	12.53	12.00	0.19	0.32	0.25	25.11	25.17	10.58	32.0	38.0	47.5	137.5	13
20	09/24/92	35.6	15.0	0.51	7.65	15.00	0.09	0.19	0.20	16.15	14.23	5.44	13.5	25.5	36.5	98.0	18
21	07/13/93	70.4	13.0	1.16	15.14	13.00	0.21	0.38	0.23	27.39	29.55	7.55	30.5	42.0	52.5	158.5	21
22	06/30/94	123.6	20.0	1.33	26.59	20.00	0.24	0.67	0.15	35.36	45.54	8.54	37.0	68.5	90.5	196.5	23
23	08/25/95	40.7	8.0	1.09	8.76	8.00	0.19	0.22	0.38	26.39	20.84	5.71	22.5	29.0	36.5	120.0	21
24	07/28/96	55.0	4.0	2.96	11.83	4.00	0.53	0.30	0.75	52.51	41.27	12.33	35.0	53.0	68.5	74.0	6
25	07/01/97	98.7	18.0	1.18	21.23	18.00	0.21	0.54	0.17	30.50	37.41	7.24	49.5	56.5	63.5	166.5	23
26	08/08/98	50.7	15.0	0.73	10.90	15.00	0.13	0.28	0.20	20.19	20.28	4.75	19.5	38.5	41.0	95.0	20
27	08/02/99	57.8	22.0	0.57	12.43	22.00	0.10	0.32	0.14	18.41	20.79	5.61	27.5	40.5	51.0	123.5	22
28	07/22/00	64.2	11.0	1.25	13.80	11.00	0.22	0.35	0.27	28.19	28.65	7.42	36.0	50.0	54.5	96.5	13
29	06/30/01	98.3	5.0	4.23	21.14	5.00	0.75	0.54	0.60	62.92	64.38	17.75	41.0	72.0	87.0	106.5	6
30	08/31/02	62.1	23.0	0.58	13.35	23.00	0.10	0.34	0.13	19.08	22.10	8.61	22.5	40.5	54.0	198.0	23
31	06/27/03	46.8	9.0	1.12	10.07	9.00	0.20	0.26	0.33	26.25	22.71	8.17	15.0	30.0	42.5	122.5	15
32	08/18/04	33.0	21.0	0.34	7.09	21.00	0.06	0.18	0.14	12.76	11.99	3.73	12.5	23.0	31.0	89.5	24
33	07/11/05	33.6	10.0	0.72	7.22	10.00	0.13	0.18	0.30	20.39	15.58	5.74	23.0	33.0	44.0	109.0	19
34	07/16/06	67.5	15.0	0.97	14.51	15.00	0.17	0.37	0.20	24.66	26.99	8.46	22.5	42.0	54.5	203.0	24
35	08/05/07	183.3	7.0	5.63	39.41	7.00	1.00	1.00	0.43	80.95	100.00	18.65	68.0	122.5	149.0	186.5	10
36	07/24/08	70.6	19.0	0.80	15.18	19.00	0.14	0.39	0.16	22.83	26.35	4.02	49.0	63.0	68.0	96.5	24
average		59.9	12.6	1.36	12.88	12.56	0.24	0.33	0.33	29.83	28.40	7.72	28.52	44.70	55.43	123.76	17.50
maximum		183.3	24.0	5.63	39.41	24.00	1.00	1.00	1.00	80.95	100.00	18.65	68.00	122.50	149.00	228.50	24.00
minimum		14.5	3.0	0.30	3.11	3.00	0.05	0.08	0.13	12.04	9.24	3.73	10.50	16.00	24.00	40.50	6.00

**Table 2:** Regression equations between each hydrograph characteristic and each of two flash flood indexes.

	Flash Flood Index, *RF*		Flash Flood Index, *FI*	
	
	Regression Equations	*R^2^*	Regression Equations	*R^2^*
*K*	*RF* =12.869*K* +12.354	0.950	*FI* =14.131*K* + 9.209	0.861
*M*	*RF* =1.418*M* +11.569	0.428	*FI* = 2.258*M* − 0.682	0.817

**Table 3:** Regression equations between each rainfall characteristic and each of two flash flood indexes.

	Flash Flood Index, *RF*		Flash Flood Index, *FI*	
	
	Regression Equations	*R^2^*	Regression Equations	*R^2^*
*I_a_*	*RF* = 3.252 *I_a_* + 4.713	0.573	*FI* = 4.044 *I_a_* − 2.835	0.666
*R*_1_*_h_*	*RF* = 0.900 *R*_1_*_h_* + 4.166	0.512	*FI* = 1.195 *R*_1_*_h_* − 5.671	0.678
*R*_2_*_h_*	*RF* = 0.597 *R*_2_*_h_* + 3.152	0.587	*FI* = 0.830 *R*_2_*_h_* − 8.692	0.854
*R*_3_*_h_*	*RF* = 0.484 *R*_3_*_h_* + 3.008	0.577	*FI* = 0.682 *R*_3_*_h_* − 9.383	0.860
*R_t_*	*RF* = 0.081 *R_t_* + 19.755	0.061	*FI* = 0195 *R_t_* + 4.253	0.265
*D*	*RF* = 0.164*D*^2^ − 6.289*D* + 84.146	0.285	*FI* = 0.187*D*^2^ − 6.495*D* + 78.691	0.137

**Table 4:** Probability rainfall data in the Oui-mi river basin.

Duration	Depth of precipitation (mm)							
	2 yrs	3 yrs	5 yrs	10 yrs	20 yrs	50 yrs	100 yrs	200 yrs
1 hrs	40.3	46.1	52.6	60.7	68.5	78.5	92.5	99.8
3 hrs	64.3	74.1	85.1	98.9	112.1	129.3	144.1	157.1
6 hrs	88.5	102.7	118.6	138.7	158	182.8	198.0	215.9
12 hrs	119.2	141.7	166.9	198.3	228.5	267.7	282.6	311.3
18 hrs	128.7	153.5	181.1	217.1	251.9	298.3	340.4	373.1
24 hrs	136.9	164.1	194.1	232.4	268.7	316.1	388.1	431.5

**Table 5:** Huff’s cumulative rainfall curve in the Oui-mi river basin.

Cumulative time (%)		0	10	20	30	40	50	60	70	80	90	100
Cumulative Rainfall (%)	1st quartile	0.0	20.1	41.3	60.3	69.4	73.6	79.5	84.2	89.6	95.2	100.0
	2nd quartile	0.0	4.7	12.0	26.0	47.7	67.7	78.0	87.1	92.1	96.3	100.0
	3rd quartile	0.0	4.9	10.7	15.8	21.9	33.7	52.1	74.7	88.9	95.4	100.0
	4th quartile	0.0	8.6	16.7	22.2	25.0	30.4	35.0	45.2	60.0	81.7	100.0

**Table 6:** Summary of runoff and flash flood indexing characteristics for design floods along with rainfall characteristics in the Oui-mi River basin.

No	Flood Runoff Characteristics			Flood Indexing Parameters					Rainfall Characteristics		
	Design flood	Flood peak discharge	Time to peak discharge	Rising curve gradient	Peak discharge magnitude	Relative Severity Factors		Flash Flood Index	Average rainfall intensity	Total rainfall depth	Rainfall duration time
	(1)	Q_p_ (m^3^/s) (2)	T (hr) (3)	K (mm/hr^2^) (4)	M (mm/hr) (5)	RK (6)	RM (7)	FI (%) (8)	I_a_ (mm/hr) (9)	R_t_ (mm) (10)	D (hr) (11)
1	2year 1hr	25.32	2	2.72	5.45	0.48	0.14	31.09	40.3	40.3	1
2	2year 3hr	51.28	3	3.68	11.03	0.65	0.28	46.63	21.4	64.3	3
3	2year 6hr	64.95	5	2.79	13.97	0.50	0.35	42.53	14.8	88.5	6
4	2year 12hr	60.46	9	1.44	13.00	0.26	0.33	29.32	9.9	119.2	12
5	2year 18hr	46.26	13	0.77	9.95	0.14	0.25	19.42	7.2	128.7	18
6	2year 24hr	38.67	17	0.49	8.32	0.09	0.21	14.89	5.7	136.9	24
7	3year 1hr	31.89	2	3.43	6.86	0.61	0.17	39.16	46.1	46.1	1
8	3year 3hr	64.36	3	4.61	13.84	0.82	0.35	58.53	24.7	74.1	3
9	3year 6hr	81.23	5	3.49	17.47	0.62	0.44	53.19	17.1	102.7	6
10	3year 12hr	77.06	9	1.84	16.57	0.33	0.42	37.38	11.8	141.7	12
11	3year 18hr	58.93	13	0.97	12.67	0.17	0.32	24.74	8.5	153.5	18
12	3year 24hr	49.47	17	0.63	10.64	0.11	0.27	19.05	6.8	164.1	24
13	5year 1hr	39.84	2	4.28	8.57	0.76	0.22	48.92	52.6	52.6	1
14	5year 3hr	78.77	3	5.65	16.94	1.00	0.43	71.64	28.4	85.1	3
15	5year 6hr	100.30	5	4.31	21.57	0.77	0.55	65.68	19.8	118.6	6
16	5year 12hr	96.26	9	2.30	20.70	0.41	0.53	46.69	13.9	166.9	12
17	5year 18hr	73.53	13	1.22	15.81	0.22	0.40	30.86	10.1	181.1	18
18	5year 24hr	61.68	17	0.78	13.26	0.14	0.34	23.76	8.1	194.1	24
19	10year 1hr	50.52	2	5.43	10.86	0.96	0.28	62.02	60.7	60.7	1
20	10year 3hr	100.63	3	7.21	21.64	1.28	0.55	91.52	33.0	98.9	3
21	10year 6hr	125.29	5	5.39	26.94	0.96	0.68	82.04	23.1	138.7	6
22	10year 12hr	120.78	9	2.89	25.97	0.51	0.66	58.58	16.5	198.3	12
23	10year 18hr	92.71	13	1.53	19.94	0.27	0.51	38.91	12.1	217.1	18
24	10year 24hr	77.24	17	0.98	16.61	0.17	0.42	29.75	9.7	232.4	24
25	20year 1hr	61.50	2	6.61	13.23	1.17	0.34	75.51	68.5	68.5	1
26	20year 3hr	131.32	3	9.41	28.24	1.67	0.72	119.43	37.4	112.1	3
27	20year 6hr	150.03	5	6.45	32.26	1.15	0.82	98.24	26.3	158	6
28	20year 12hr	144.50	9	3.45	31.08	0.61	0.79	70.09	19.0	228.5	12
29	20year 18hr	111.79	13	1.85	24.04	0.33	0.61	46.92	14.0	251.9	18
30	20year 24hr	92.36	17	1.17	19.86	0.21	0.50	35.57	11.2	268.7	24
31	50year 1hr	76.44	2	8.22	16.44	1.46	0.42	93.85	78.5	78.5	1
32	50year 3hr	149.45	3	10.71	32.14	1.90	0.82	135.92	43.1	129.3	3
33	50year 6hr	182.77	5	7.86	39.31	1.40	1.00	119.68	30.5	182.8	6
34	50year 12hr	175.94	9	4.20	37.84	0.75	0.96	85.34	22.3	267.7	12
35	50year 18hr	137.26	13	2.27	29.52	0.40	0.75	57.61	16.6	298.3	18
36	50year 24hr	112.14	17	1.42	24.12	0.25	0.61	43.19	13.2	316.1	24
37	100year 1hr	87.62	2	9.42	18.84	1.67	0.48	107.58	92.5	92.5	1
38	100year 3hr	174.44	3	12.50	37.51	2.22	0.95	158.65	48.0	144.1	3
39	100year 6hr	202.91	5	8.73	43.64	1.55	1.11	132.87	33.0	198.0	6
40	100year 12hr	197.66	9	4.72	42.51	0.84	1.08	95.87	23.6	282.6	12
41	100year 18hr	156.28	13	2.59	33.61	0.46	0.85	65.60	18.9	340.4	18
42	100year 24hr	130.13	17	1.65	27.98	0.29	0.71	50.12	16.2	388.1	24
43	200year 1hr	98.61	2	10.60	21.21	1.88	0.54	121.07	99.8	99.8	1
44	200year 3hr	196.90	3	14.11	42.34	2.51	1.07	179.07	52.4	157.1	3
45	200year 6hr	227.09	5	9.77	48.84	1.73	1.24	148.70	36.0	215.9	6
46	200year 12hr	220.53	9	5.27	47.43	0.94	1.20	106.97	25.9	311.3	12
47	200year 18hr	174.35	13	2.88	37.49	0.51	0.95	73.18	20.7	373.1	18
48	200year 24hr	145.34	17	1.84	31.26	0.33	0.79	55.98	18.0	431.5	24
average		108.43	8.17	4.51	23.32	0.80	0.59	69.65	27.18	174.99	10.67
maximum		227.09	17	14.11	48.84	2.51	1.24	179.07	78.50	316.10	24
minimum		31.89	2	0.49	6.68	0.09	0.17	14.89	5.70	46.10	1
recorded max.		183.26	24	5.63	39.41	1.00	1.00	100.00	18.65	228.50	24
